# Modern Himalayan marmot faeces analyses: Implications for palaeodietary, palaeoecological, and palaeoclimate analysis

**DOI:** 10.1371/journal.pone.0358642

**Published:** 2026-10-28

**Authors:** Sadhan K. Basumatary, Hukam Singh, Manish Mehta, Swati Tripathi, Mahesh G. Thakkar, H. Gregory McDonald, Eline N. van Asperen, Vinit Kumar

**Affiliations:** 1 Birbal Sahni Institute of Palaeosciences, Lucknow, Uttar Pradesh, India; 2 Wadia Institute of Himalayan Geology, Dehradun, India; 3 Department of Earth Science, Denver Museum of Nature and Science, Denver, United States of America; 4 School of History, Classics and Archaeology, Newcastle University, Newcastle upon Tyne, United Kingdom; 5 Department of Geology, University of Lucknow, Lucknow, Uttar Pradesh, India; National Cheng Kung University, TAIWAN

## Abstract

Pollen and nutrient analyses of the faeces of the Himalayan marmot (*Marmota himalayana*) were made to understand its preferred dietary plants and ecology. Pollen preserved in the faeces accurately reflected its diet as a mixed feeder including *Artemisia*, Caryophyllaceae, Asteroideae, and *Ephedra*, characteristic plants of the Alpine steppe vegetation under a cold and arid climate in the higher Himalaya. Pollen ratio values between *Artemisia*/Chenopodiaceae, *Artemisia*/Cyperaceae, and *Artemisia*/*Ephedra* are identified from the Pensilla Pass and Rimkhim regions, as useful tools that characterize the Alpine steppe vegetation and climate in the higher Himalaya. Principal Component Analysis of the pollen taxa indicated only a slight difference in the relative abundance between the two localities sampled, Pensila Pass and Rimkhim areas. Coprophilous fungal spores, *Sporormiella* and *Saccobolus*, were abundant and considered useful for understanding the interrelationship between vegetation and the marmot’s preferred habitat. Fifteen nutritional elements were recorded, and the high concentration of carbon, nitrogen, calcium, silicon, and, phosphorous is considered significant with a direct dietary relationship with the preferred plants consumed. This study provides a modern analogue and a baseline for coprolite and palaeoecological analyses in marmot inhabited regions and to observe the species future response to vegetation and climate change resulting shifts in altitude in the higher Himalaya.

## Introduction

The Himalaya encompasses the highest mountain ranges compared to the adjacent Tibetan plateau which spans roughly 3 million km^2^ with an average altitude exceeding five kilometres [1]. Due to this vast and elevated terrain, the region is often referred to as the ‘Third Pole’ [2], as it significantly influences global climate systems through its extensive orography. This region plays a vital role in shaping the East Asian and South Asian summer monsoons [3]. Geological records suggest the Himalaya uplift began approximately 50–55 million years ago, following the collision between the Indian and Eurasian plates [4]. This uplift continued into the Pleistocene and, contributed to the formation of widespread mountain glaciations. Today, the Himalaya is recognized as a biodiversity hotspot and a key region for studying environmental changes across time. Its ecosystems, highly sensitive to climate variations, offer valuable insights into both natural and human-induced changes [5]. Monitoring the current biodiversity patterns along latitudinal and altitudinal variation in the Himalaya can reveal how climate change influences the distribution of plant and animal species [6].

The pollen-based vegetation studies have been the key target of palaeoecological research [7–9] in relation to the different environmental condition. The data derived from coprolites have been less frequently employed compared to analyses of lake sediments and peat bogs, largely because coprolite preservation is relatively uncommon [10]. Exceptions are found in arid regions, where dry cave environments facilitate their preservation [11–13]. Despite their rarity, herbivore coprolites are valuable as they contain plant remains that offer direct insights into the dietary preferences of extinct species and their associated habitats [14–17]. Such studies are especially significant in arid landscapes where traditional sites of pollen preservation are poorly developed or entirely absent [10,18]. For instance, coprolites analyses have yielded direct evidence of past diet and environments in the southwestern United States [19,20]. Moreover, in the higher Himalayan regions especially Ladakh, the quality of vegetation is poor under arid conditions and the analysis of the herbivores dung can serve as a supplementary substrate to the soil sample for modern palynological analysis.

The presence, decline, and disappearance of coprophilous fungal spores within sedimentary records can serve as valuable indicators of herbivore populations, shedding light on fluctuations in population size and extinctions in response to changes in the vegetation and climate [21]. Palynological analyses of modern herbivore dung have been conducted to identify dietary plants in relation to local vegetation and climate conditions [22–26]. These studies have suggested the importance of dung and coprolites as records of palaeoecological and palaeoherbivory research [10,24,27]. The dynamic relationship between herbivores and the plants they consume is closely influenced by factors such as altitude, latitude, vegetation type, and regional climate parameters such as temperature and precipitation. Many plant and animal species are highly sensitive to changes in environmental conditions, often exhibiting restricted and patchy distributions. The distribution of *Marmota himalayana,* commonly known as the Himalayan marmot, is found throughout the Himalayan ranges of India, Nepal, and Pakistan. This species is currently listed as endangered on the International Union for Conservation of Nature (IUCN) Red list of Threatened species [28]. Therefore, the identification and characterization of the relationship between this species and vegetation type in mountainous regions is crucial for environmental in relation to the modern faeces and coprolite research. This applies not only to larger species but also to small mammals, which can play essential ecological roles in these sensitive ecosystems [5,29,30].

In this study, we present the analysis of faeces of the Himalayan marmot from two study sites in the Indian Himalayas (Pensila Pass and Rimkhim) to identify its preferred dietary plants from the surrounding vegetation and to assess the potential of pollen and spore assemblages preserved in its faeces for reconstructing past plant distributions. This method also offers insights into how the Himalayan marmot and other alpine species might respond to future climate changes. Through the analysis of the marmot faeces, we aim to gain direct insights into the vegetation on which marmot rely, thereby improving our understanding of their ecological requirements. This research provides valuable new records on pollen and dung loving fungal diversity in the extreme environment from a small alpine mammal.

## Study area, vegetation, and wildlife

The research was conducted at two high-altitude sites in the higher western Himalaya. The first site is situated at Pensila Pass in the Suru River basin, Zanskar region of Ladakh. The second site is located in the Rimkhim area of the Dhauliganga Valley in the Uttarakhand Himalaya (Fig 1).

**Fig 1:**
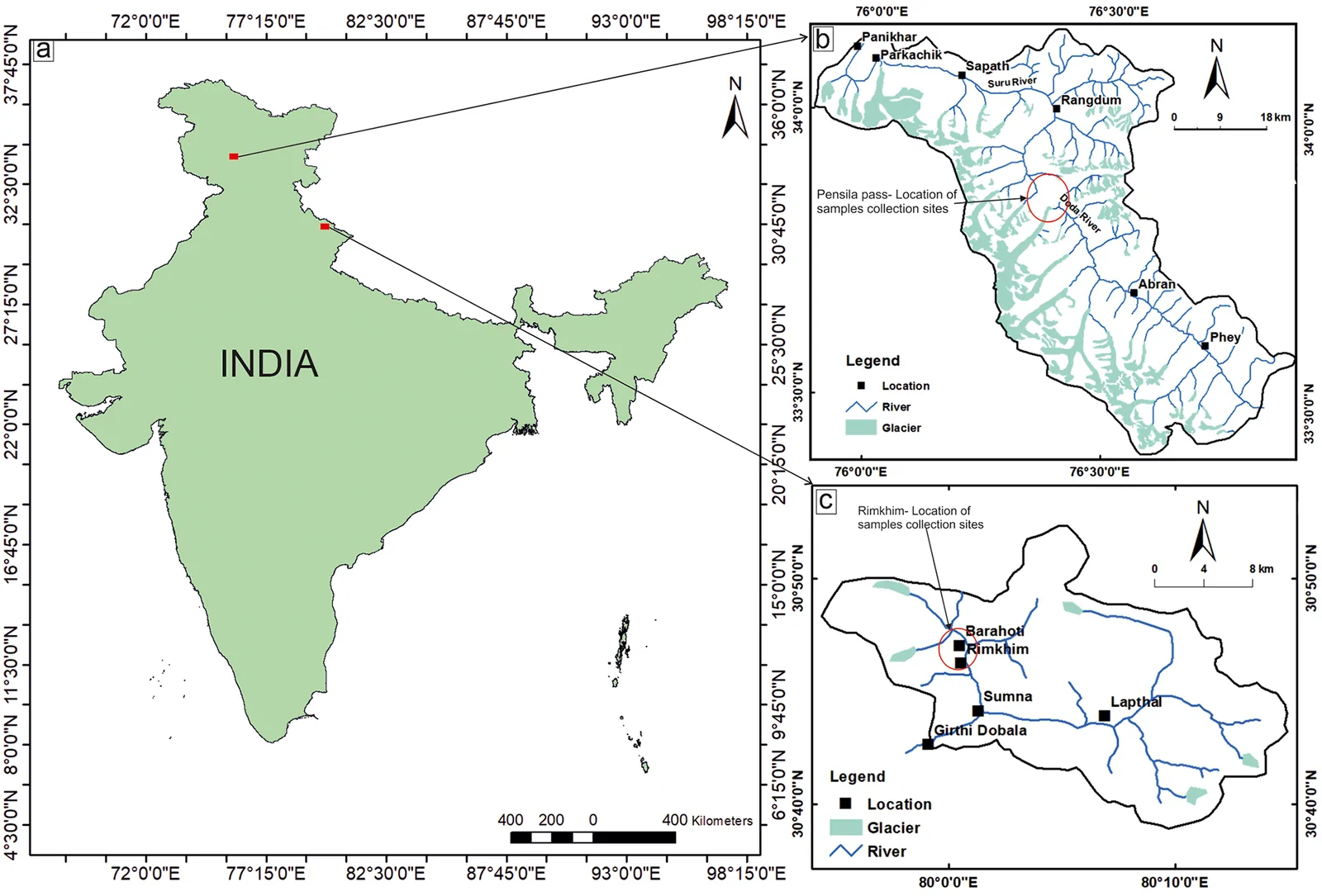
Study areas. a. Regional map showing the sampling locations, b. Close up of the sample collection site (Pensila Pass), c. Close up of the sample collection site (Rimkhim area).

The altitude of the sampling sites ranges from 4000–4200 m above sea level (asl) in the Suru River basin and from 3600–4000 m asl in the Dhauliganga basin, in the high-altitude regions of the Himalayas. Pensila Pass spans approximately 2 km^2^, featuring gentle slopes between 2^o^ and 5^o^. These favourable topographic conditions, along with a consistent water supply, support the presence of hundreds of marmot colonies [31].

The vegetation of the Pensila Pass is primarily Alpine steppe, dominated by plant families such as Poaceae, Cyperaceae, Asteraceae, Polygonaceae, Fabaceae, Liliaceae, Papaveraceae, Onagraceae, Rosaceae, Chenopodiaceae, Amaranthaceae, Caryophyllaceae, and Ranunculaceae. Some common plant species include *Artemisia brevifolia* Wall. ex DC., *Cousinia thomsonii* C. B. Clarke, *Carex melanantha* C.A. Mey., *Caragana versicolor* Benth., *Stipa orientalis* C.A. Mey., *Potentilla rigida* Wall. ex Lehm., *Ephedra gerardiana* Wall. ex-Florin, and *Lonicera spinosa* Jacquem. ex Decne [32,33].

Similarly, the Rimkhim area covers about 1 km^2^ exhibits gentle slopes, with several marmot colonies recorded there [28]. These high-altitude zones of western Himalaya are characterized by cold desert conditions [34,35]. The vegetation of the study area is primarily Alpine steppe, and commonly growing plant families includes Poaceae, Cyperaceae, Asteraceae, Polygonaceae, Fabaceae, Liliaceae, Papaveraceae, Onagraceae, Rosaceae, Chenopodiaceae, Amaranthaceae, Caryophyllaceae and Ranunculaceae. Common species such as *Artemisia brevifolia* Wall. ex DC., *Carex melanantha* C.A.Mey., *Caragana versicolor* Benth, *Stipa orientalis* C.A.Mey., and *Lonicera spinosa* Jacquem.ex Decne are abundant. The herbaceous flora is interspersed with scattered shrubby elements such as *Juniperus squamata* D. Don., *J. indica* Bertol., *Rosa macrophylla* Lindl., and *Ephedra gerardiana* Wall. ex-Florin.

In addition to *Marmota himalayana*, the region is home to a diverse assemblage of large mammals, including *Bos mutus* (wild yak), *Capra sibirica* (ibex), *Pseudois nayaur* (blue sheep), *Equus kiang* (Tibetan wild ass), *Ursus arctos* (bear), *Uncia uncia* (snow leopard), and *Canis lupus chanco* (Tibetan wolf). The area also supports several smaller herbivorous mammals such as *Ochotona curzoniae* (pika), *Lepus oiostolus* (plateau hare), and *Eospalax baileyi* (plateau zokor) [36] (Fig 2a-g).

**Fig 2:**
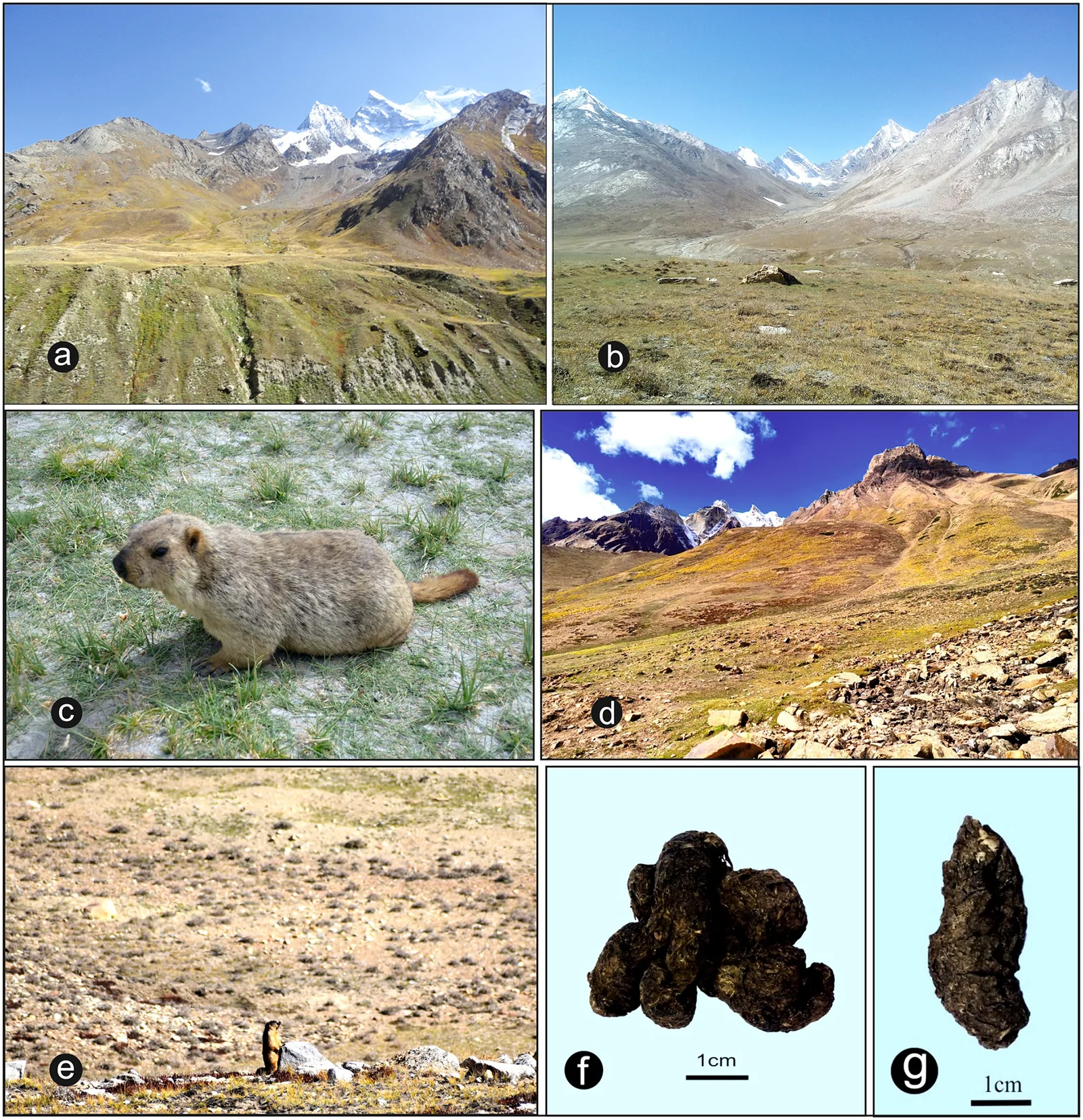
Field photographs. a and b. Alpine steppe vegetation at Pensila Pass in the Himalayan marmot habitat area. c. A Himalayan marmot in their natural habitat in Pensila Pass area. d. Alpine steppe vegetation in Rimkhim area. e. A Himalayan marmot in natural habitat in Rimkhim area. f. Midden faeces of *Marmota himalayana*, g. Shape and size of the marmot’s faeces pellet. Photograph credits by Hukam Singh, Manish Mehta, and Vinit Kumar.

### Climate and soil

The regional climate is characterized by the extreme cold and aridity [33]. Summer temperature can occasionally rise above 25° C, while winter temperature may decline to as low as −40° C [37]. The primary source of moisture in the study area are the mid-latitude westerlies and to a lesser extent Indian Summer Monsoon. The high-altitude and mid-latitude westerlies predominantly influenced precipitation and these areas often remain snow-covered for most of the year, with winter snowfall occasionally exceeding 5 m. Due to their location in the rain shadow zone, the regions receive minimal summer precipitation, often limited to just 25–300 mm/year, as they are largely unaffected by the Indian Summer Monsoon [37]. Along with the low and irregular rainfall, the areas are also subject to strong winds throughout the year. The prevailing soil types in the study sites are silty and loamy clay.

## Materials and methods

### Sampling

Between August and September 2023, 25 faeces samples (P1-P25) from marmot habitats were collected at Pensila Pass, coinciding with the region’s peak flowering season. The samples were gathered at roughly 25 m intervals to help identify the marmot’s preferred dietary plants within this habitat. Additionally, 20 faeces samples (R26-R45) from the Rimkhim area were collected during the same months. Each faeces sample include 3–5 pellets with preference given to samples from adult individuals based on the size and morphology, when possible (Fig 2f and 2g). However, some samples may represent multiple individuals, as suggested by variations in pellet size. A few samples were identified as originating from juvenile marmots based on their smaller size and morphology. The collected faeces were 3–5 days old and semi-dry when collected and were stored separately in zip lock polythene bags to prevent contamination before laboratory analysis.

### Laboratory analysis

To minimize the risk of contamination from airborne palynomorphs, the outer surface of each faeces pellet was carefully removed using a sterilized knife. Approximately 50–80 g of faeces material was removed from the inner portion, ground thoroughly to create a homogeneous mixture, and 30 g removed for analysis. The samples were processed for pollen and non-pollen palynomorphs (NPPs) following a standard acetolysis method [38]. This involved sequential treatments with 10% aqueous potassium hydroxide (KOH) solution to deflocculate the material, 40% hydrofluoric acid (HF) to dissolve siliceous content, followed by glacial acetic acid (GAA). Acetolysis was then performed using a mixture of acetic anhydride and concentrated sulfuric acid (H_2_SO_4_) in a 9:1 ratio to remove cellulose. Subsequently, samples were rinsed twice with GAA and 3–4 times with distilled water. The processed material was sieved through 500 µm mesh and preserved in a 50% glycerol solution containing a few drops of phenol to protect them against microbial decomposition. From each sample, between 245–284 pollen grains and NPPs, including *Arcella* and *Botryococcus* were identified, counted and the frequency calculated to make a pollen frequency diagram (Fig 3).

**Fig 3:**
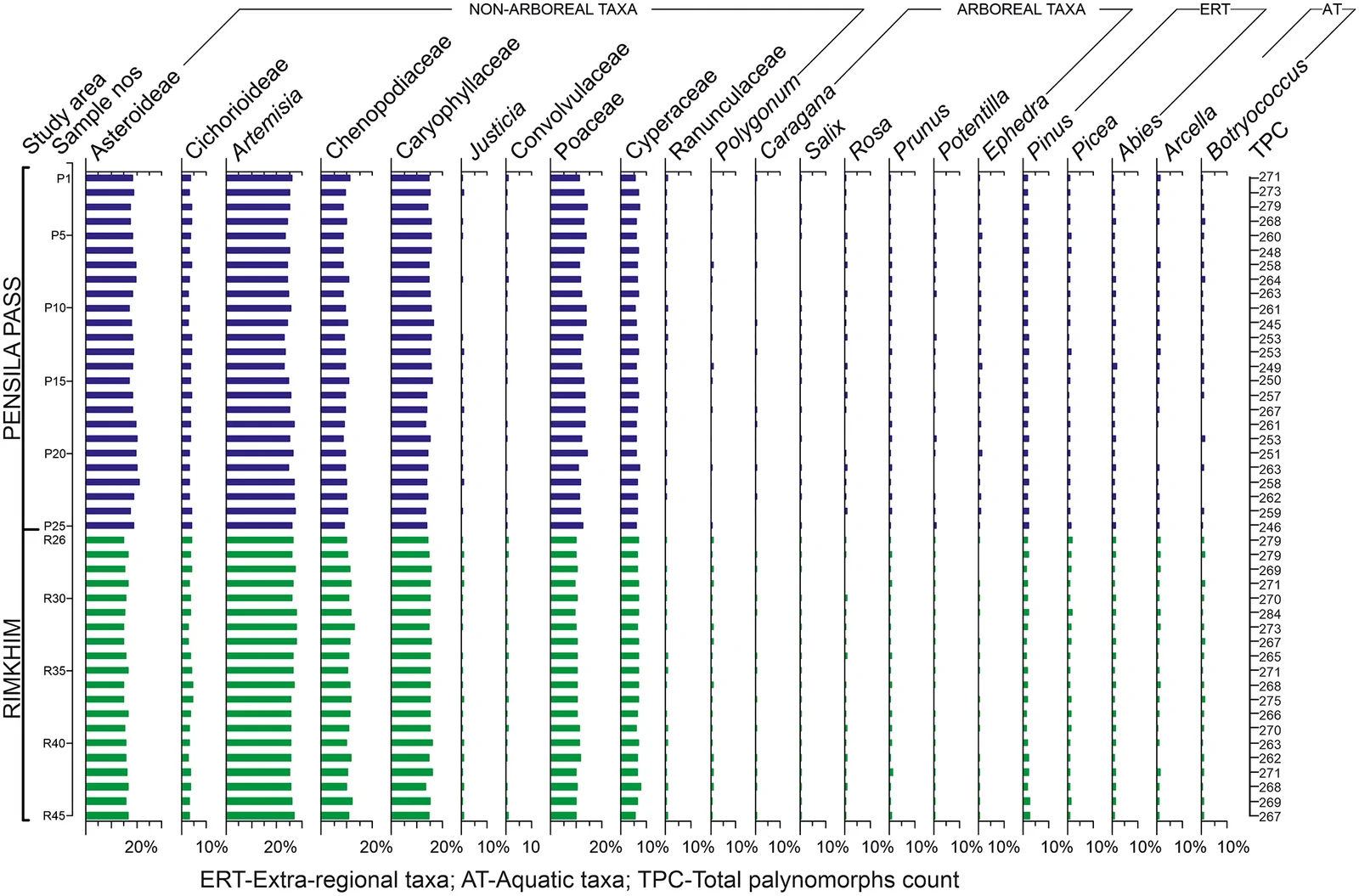
Pollen frequency diagram from the Pensila Pass and Rimkhim area.

The recovered pollen and NPPs were classified into four categories: arboreal taxa, non-arboreal taxa, extra-local taxa, and aquatic taxa. Fungal spores were analysed separately, with counts ranging from 212 to 265 per palynological slide to make the fungal spore spectra (Fig 4).

**Fig 4:**
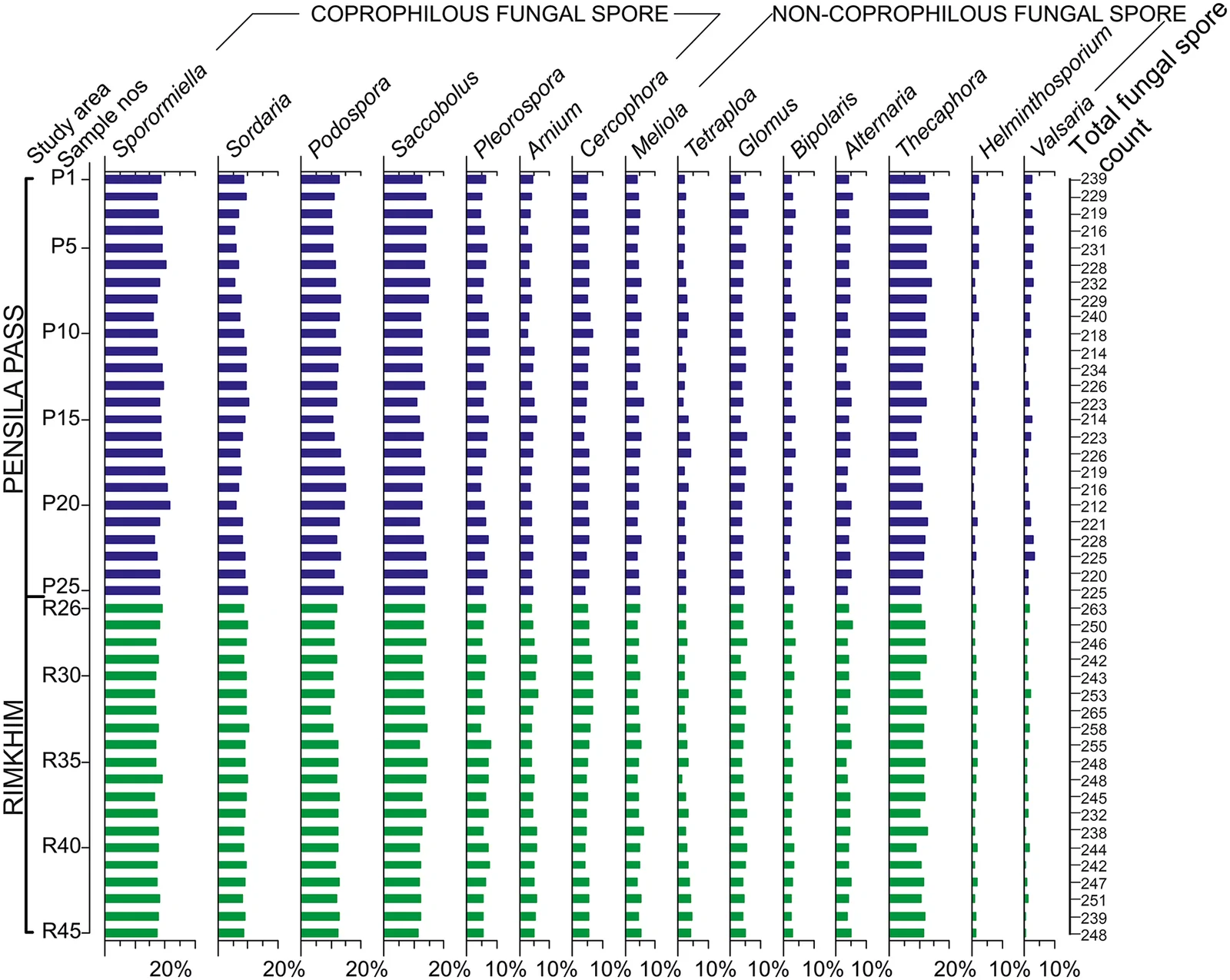
Fungal spore frequency diagram from the Pensila Pass and Rimkhim area.

The fungal spores were further categorized as coprophilous and non-coprophilous fungal spores respectively. For the identification of pollen grains and fungal spores, reference slides from the herbarium of the Birbal Sahni Institute of Palaeosciences (BSIP) Lucknow (India) were consulted alongside published literature and photographic records [25,39]. Microphotographs were taken using an Olympus BX61 × 40X with a DP-25 digital camera (Fig 5).

**Fig 5:**
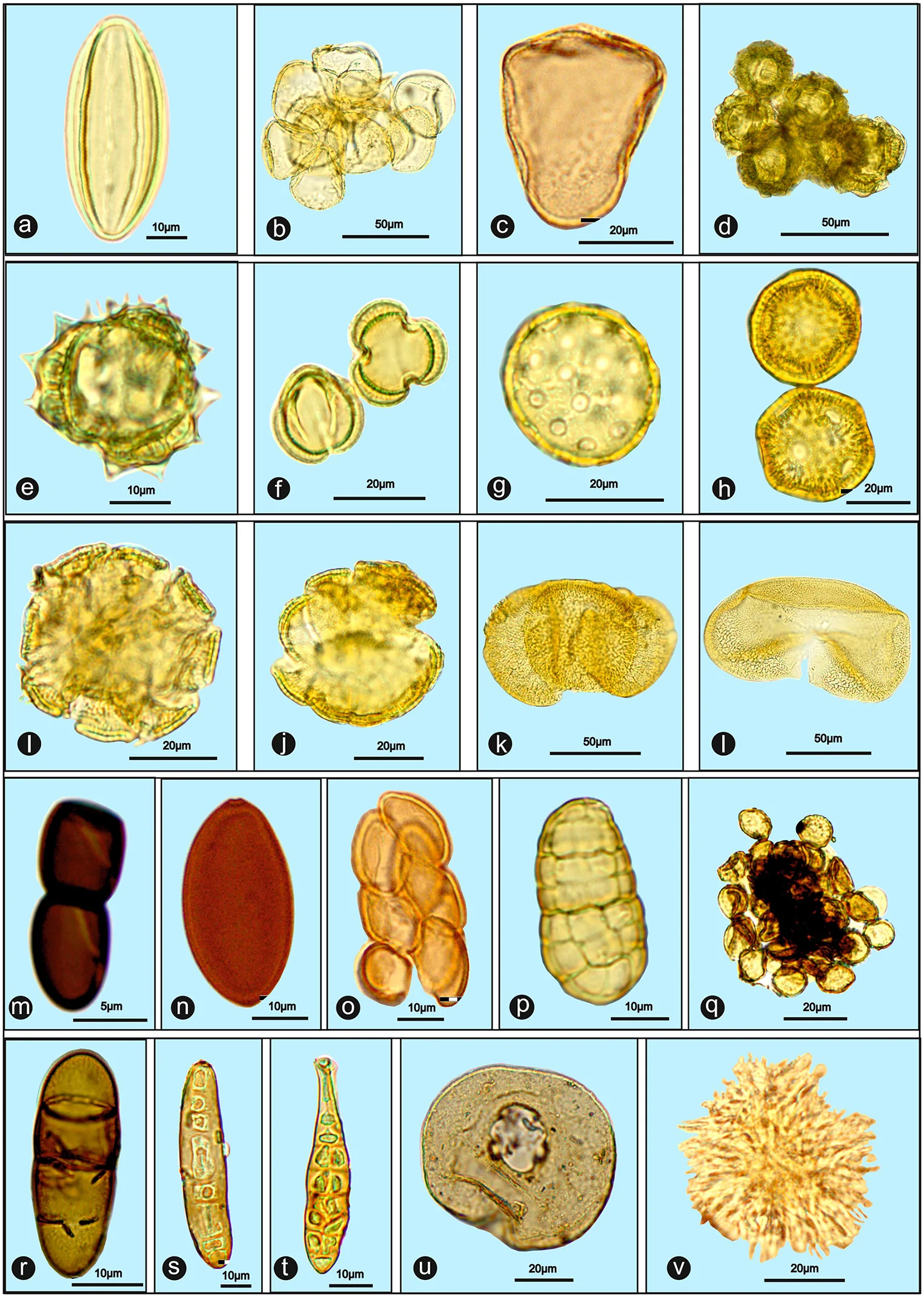
Palynomorphs assemblage recovered from the *Marmota himalayana* faeces samples. a. *Ephedra*, b. Cluster of Poaceae pollen, c. Cyperaceae, d. Cluster of Asteroideae pollen, e. Asteroideae, f. *Artemisia*, g. Chenopodiaceae, h. Amaranthaceae, i. and j. Convolvulaceae, k. *Pinus* sp., l. *Abies*, m. *Sporormiella*, n. *Podospora*, o. Cluster of *Saccobolus*, p. *Pleospora*, q. Cluster of *Thecaphora*, r. *Meliola*, s. *Bipolaris*, t. *Alternaria*, u. *Arcella*, v. *Botryococcus*.

### Principal Component Analysis (PCA) analysis

Principal Component Analysis (PCA) was conducted on the pollen assemblages to assess the potential differences in dietary plants of the Himalayan marmot between the two study (Pensilla Pass and Rimkhim) regions. The statistical significance of the quantified data of pollen frequency obtained from the faeces samples was determined by SPSS 11.5.0, software, USA [40]. A probability of *p*-value ≤ 0.05 was taken to indicate statistical significance. The resulting data were imported into Unscrambler X Software package (Version 10.0.1, CAMO, USA) for multivariate unsupervised PCA (Fig 6).

**Fig 6:**
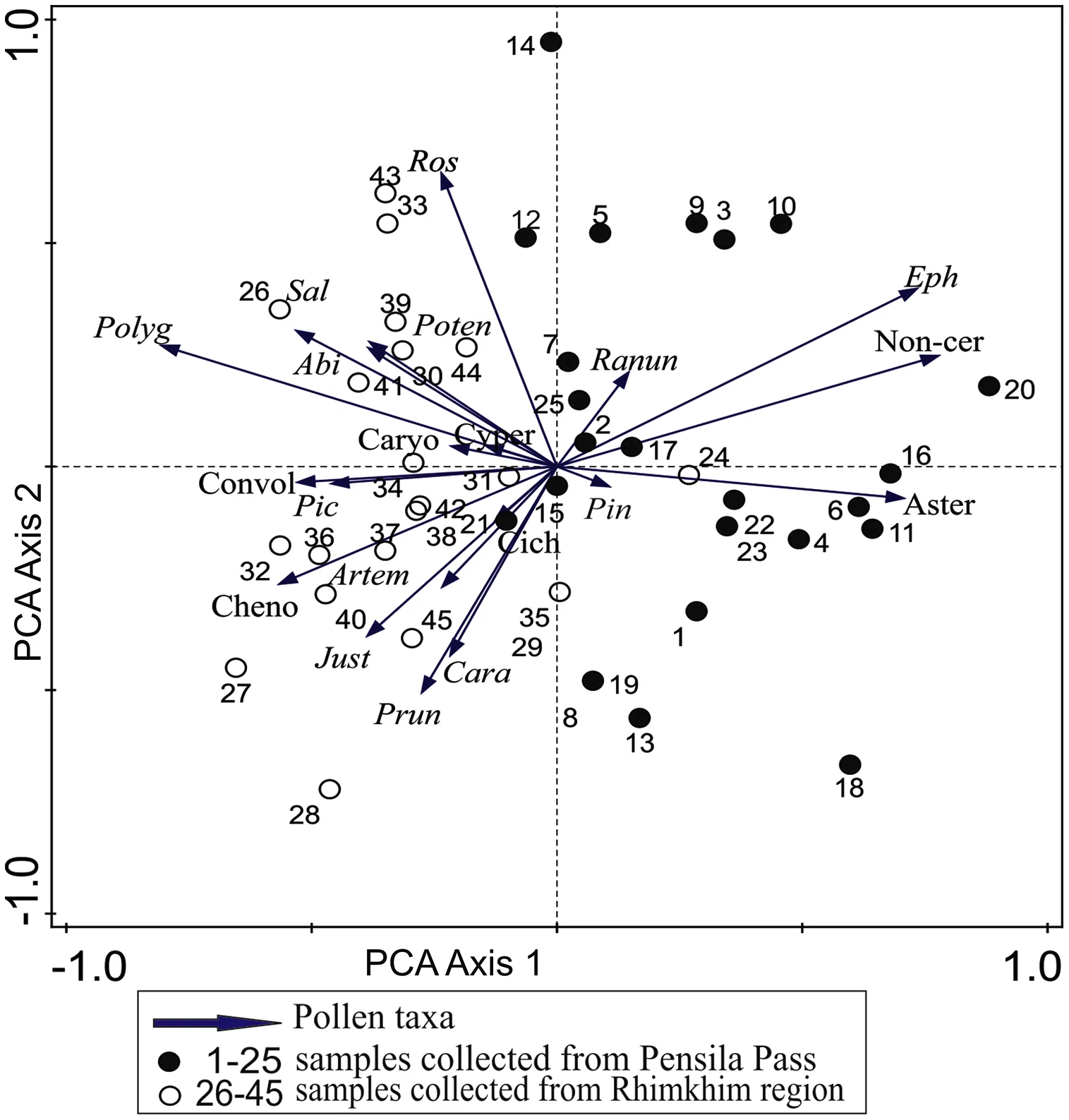
Principal Component Analysis (PCA) analysis of the pollen assemblages of *Marmota himalayana* faeces between Pensila Pass and Rimkhim area. Full form of acronym used in the figure is provided below: Ros: *Rosa*; Eph: *Ephedra*; Caryo: Caryophyllaceae; Cyper: Cyperaceae; Abi: *Abies*; Polyg: Polygonaceae; Poten: *Potentilla*; Sal: *Salix*; Ranun: Ranunculaceae; Non-cer: Non-cereal; Aster: Asteroideae; Pin: *Pinus*; Convol: Convolvulaceae; Pic: *Picea*; Artem: *Artemisia*; Cheno: Chenopodiaceae; Just: *Justicia*; Prun: *Prunus*; Car: *Caragana*; Cich: Cichorioideae.

### Field Emission Scanning Electron Microscope (FESEM-EDS) nutrient analysis

This analysis was used to identify and quantify the nutrient composition of Himalayan marmot faeces from both study areas (S1 Fig 1; S1 Table 1 and S2 Table 2). The entire set of 45 samples from both regions was analysed by FESEM-EDS. The prepared samples were first cleaned using an ultrasonicator to prevent clumping and ensure proper dispersion for nutrient analysis. After preparation the samples were mounted onto stubs for analysis. Field Emission Scanning Electron Microscopy (FESEM) combined with Energy Dispersive Spectroscopy (EDS) was performed using a JEOL, JSM-7610F FESEM system coupled with an EDAX (USA) EDS system, operating at 25 keV.

## Results

### Pollen and fungal spore data

#### Pensila Pass region.

The palynological analysis of 25 faeces samples revealed a predominance of non-arboreal taxa, including Asteroideae, Cyperaceae, Chenopodiaceae, *Artemisia*, and *Justicia*, present in proportion ranging from 0.4%− 27.4% in the pollen assemblages. Arboreal taxa, represented by *Ephedra*, *Potentilla*, *Prunus*, and *Salix*, are consistently present, though in lower abundances (0.4% −1.2%). Additionally, extra-local arboreal taxa including *Pinus*, *Cedrus*, and *Abies* were recorded with frequencies between 0.4%−2.4%. Other palymorphs such as *Arcella* and *Botryococcus* each ranging from 0.4%−1.2% were also detected (Fig 3).

Fungal spores were recovered from the same palynological slides and indicated a clear dominance of coprophilous over non-coprophilous types, comprising 67.1% and 32.9% of the assemblage respectively. Among the coprophilous fungal spores, *Sporormiella*, *Sordaria*, and *Podospora* were most abundant ranging from 4.4%−20.2%. Non-coprophilous fungal spores, primarily *Thecaphora*, *Meliola*, *Glomus*, and *Tetraploa*, were also consistently present with values ranging between 0.4%−13.9% (Fig 4).

#### Rimkhim region.

The palynological analysis of 20 faeces samples from Rimkhim area revealed the presence of Asteroideae, Cyperaceae, Chenopodiaceae, *Artemisia*, and *Justicia*, in the pollen assemblages with concentration ranging from 0.4%−27.7%. Arboreal taxa, such as *Ephedra*, *Potentilla*, *Prunus*, and *Salix*, were consistently represented with frequencies between 0.4%−1.5%. Extra-local arboreal taxa, including *Pinus*, *Cedrus*, and *Abies*, were also present with values ranging from 0.7%−2.6%. *Arcella* and *Botryococcus* were identified in low abundances each ranging from 0.4%−1.1% (Fig 3).

In the fungal spore analysis coprophilous fungal spores were more abundant than non-coprophilous ones, comprising 67.6% and 32.4% of the total count, respectively. Among the coprophilous fungal spores, *Sporormiella*, *Saccobolus*, *Sordaria*, and *Podospora* were prominent, with each taxa ranging between 3.8%−19.0%. Non-coprophilous fungal spores were mainly represented by *Meliola*, *Glomus*, and *Tetraploa,* with frequencies between 0.4%−12.6% in the assemblages (Fig 4).

### FESEM-EDS nutrient analysis

The FESEM-EDS elemental analysis of the faeces samples from the Pensila Pass region revealed the following composition: Oxygen at 45.74% by weight, followed by Carbon at 20.11%, Nitrogen at 7.78%, Calcium at 8.48%, Silicon at 4.23%, Phosphorus at 3.82%, and Potassium at 3.74%.

In comparison, the FESEM-EDS elemental analysis of the marmot faeces samples from Rimkhim area showed a slightly higher Oxygen content at 48.71% by weight, followed by Carbon at 24.68%, Calcium at 6.84%, Nitrogen at 4.75%, Phosphorus at 4.11%, Silicon at 2.71%, and Potassium at 1.94% (S1 Fig 1; S1 Table 1 and S2 Table 2).

## Discussion

### Dietary plants in relation to the vegetation and climate

Analysis of pollen assemblages from faeces samples collected at Pensila Pass and Rimkhim indicates Himalayan marmots have a preference for a mix of plants in their diet in relation to the available Alpine steppe vegetation found in the cold and arid climate in the higher Himalaya. Among dietary plants, the abundance of *Artemisia*, Caryophyllaceae, Asteroideae, Poaceae, Cyperaceae, and Chenopodiaceae pollen in the assemblages are identified and are characterised as the primary dietary plants necessary for their survival in the cold and arid climate of the Alpine steppe vegetation of the higher Himalaya.

The frequent occurrences of pollen clusters, especially Caryophyllaceae, *Polygonum*, Asteroideae, Poaceae, and *Artemisia*, in the faeces suggests these plants are locally sourced and likely consumed directly through the ingestion of flowers. These clusters are indicative of the season when these plants are in bloom and are consumed [25,41].

The frequent occurrence of *Salix*, *Potentilla*, and *Prunus* pollen, along with *Arcella* and *Botryococcus* in the pollen assemblages, strongly suggests the presence of perennial water system in and around the study areas. *Salix*, *Potentilla*, and *Prunus* typically thrive along water courses and in moist habitats [34,42]. The presence of *Arcella* and *Botryococcus* in the faeces likely results from the marmots ingesting water from these local sources. This interpretation is further supported by previous research indicating that 86% of Himalayan marmot burrows are located within 700 m of water bodies such as streams, rivers, or ponds [43]. The *Artemisia*-Caryophyllaceae-Chenopodiaceae-Asteroideae pollen assemblage is characterized in the faeces samples in relation to the Alpine steppe vegetation which accurately matches with the surveyed vegetation type [33,44]. In the context of our study, the sites situated at altitude between 3600–4200 m asl and the pollen assemblages accurately reflect the local flora corresponding to the altitude range typically inhabited by Himalayan marmots (approximately 3900–5200 m asl).

Notably, not all pollen found in the faeces originates from local sources. The presence of pollen from the extra-local taxa namely *Pinus*, *Picea*, and *Abies* is likely due to anemophilous (wind-pollinated) transport from lower altitudes. Their occurrence in the high-altitude pollen spectra suggests significant upthermic wind activity in the study areas [45,46]. The region experiences dry climate conditions that can generate strong winds, sometimes reaching velocities of 50–60 km/h. Although, in theory, dietary plants can be inferred based on the high frequency of their pollen [47], this becomes problematic in regions where wind-pollinated taxa dominate the pollen assemblages due to their high productivity and long distance transport. Additionally, some pollen grains may enter the faeces indirectly either adhered to consumed plant surfaces or present in drinking water [47,48]. So, in that case the local vegetation with their abundance of pollen taxa in the faeces samples must be considered to trace the preferred dietary plants in relation to the current vegetation and climate in the marmot habitat, rather than from extra-local sources region. A list of the major pollen taxa of the dietary plants in relation to the vegetation and their ecological significance is given in the Table 1.

**Table 1:** Characterization of major dietary pollen taxa in relation to the vegetation and their ecological significance.

Pollen taxa	Major dietary plant and ecological significance
*Artemisia*	Locally sourced and likely consumed by flowers ingestion. Alpine steppe vegetation (cold and arid conditions). Indicator of glacial and interglacial periods.
Caryophyllaceae	Locally sourced and likely consumed by flowers ingestion. Alpine steppe vegetation (cold and arid conditions).
Asteroideae	Locally sourced and likely consumed by flowers ingestion. Alpine steppe vegetation (cold and arid conditions).
Poaceae	Locally sourced and likely consumed by flowers ingestion. Alpine steppe vegetation (cold and arid conditions).
Cyperaceae	Locally sourced and likely consumed by flowers ingestion. Presence of perennial water system in and around the study areas.
Chenopodiaceae	Alpine steppe vegetation (cold and arid conditions)
*Polygonum*	Locally sourced and likely consumed by flowers ingestion. Presence of perennial water system in and around the study areas.
*Rosa*	Alpine steppe vegetation (cold and arid conditions)
Ranunculaceae, Convolvulaceae	Locally sourced and likely consumed by flowers ingestion. Presence of perennial water system.
*Potentilla*	Perennial water system in and around the study areas.
*Ephedra*	Alpine steppe vegetation (cold and arid conditions).
*Salix*	Alpine steppe vegetation (cold and arid conditions). Perennial water system in and around the study areas.
*Caragana*	Alpine steppe vegetation (cold and arid conditions).
*Prunus*	Perennial water system in and around the study areas.
*Pinus*	Anemophilous pollination, Wind-dispersed pollen from lower altitude. Indicator of upthermic wind activity
*Picea*, *Abies*	Wind-dispersed pollen from lower altitude. Indicator of upthermic wind activity

### Fungal spore preservation and its implication for coprolite and palaeoherbivory analysis

It is widely recognized that animal dung is a reliable source of palaeomycological data [14]. Among the fungal spores, both coprophilous and non-coprophilous, such as *Sporormiella*, *Podospora*, *Saccobolus*, *Helminthosporium*, and *Meliola* are consistently present in the faeces of both areas sampled. The clustering of *Sporormiella*, *Podospora*, and *Saccobolus* in the assemblages of the studied samples strongly suggest a local origin due to their short dispersal capacity [49]. In our samples, the coprophilous fungal spores are more abundant than non-coprophilous fungal spore. Among these, *Sporormiella* is the most dominant with occurrences of 18.5% and 17.5% in the Pensila Pass and Rimkhim area respectively. Other frequently encountered coprophilous spores include *Saccobolus* and *Podospora*, which, along with *Sporormiella*, consistently appear at high frequencies (ranging from 9.8% to 21.7%) in the marmot faeces. This composition closely resembles fungal spore assemblages identified in the dung of other herbivores such as rhinoceros, wild yak, red panda, and wild ass [25,26]. The recovery of the major fungal spores from the marmot faeces samples with their significant inferences are listed in the Table 2.

**Table 2:** Characterization of major fungal spore in relation to their source and ecological significance.

Major Fungal spore	Habitat/Ecological significance
*Sporormiella*	Coprophilous fungi; Local original; Indicator of herbivore activity
*Podospora*	Coprophilous fungi; Local original; Indicator of herbivore activity
*Saccobolus*	Coprophilous fungi; Local original; Indicator of herbivore activity
*Sodaria*	Coprophilous fungi; Local original; Indicator of herbivore activity
*Thecaphora*,	Pathogenic; Consumption of infected *Silene* (Caryophyllaceae) plants
*Helminthosporium*	Pathogenic; Consumption of infected Poaceae, and Cyperaceae
*Alternaria*,	Pathogenic; Consumption of infected Poaceae, and Cyperaceae
*Tetraploa*	Pathogenic; Consumption of infected dietary plants
*Meliola*	Pathogenic; Consumption of infected dietary plants

The presence and relative abundance of the coprophilous fungal spore *Sporormiella* in the soil and other sediments is widely used as an indicator of herbivore population sizes and activity [50,51]. While this spore is typically associated with megaherbivores [51], this study demonstrates that *Sporormiella*, along with other coprophilous fungal spores, *Saccobolus* and *Podospora*, can also occur in the faeces of smaller herbivores such as the Himalayan marmot. This is consistent with earlier studies on extant small mammals, which have documented the growth of dung fungi on the faeces of lagomorphs, rodents, and even birds [52–55]. Notably, *Sporormiella* and *Sordaria* have also been identified in Pleistocene midden dung of the Arctic ground squirrel (*Spermophilus parryii*) [14]. *Sporormiella* spores in soil are of local origin as its active dispersal radius is 1 m or less [21,49], although airborne transport does occur resulting in dispersal up to 100 m from the dung source [51]. However, the coprophilous fungal spore, *Sporormiella*, *Sordaria*, and *Saccobolus* preserve well in the faeces sample within the foraging zone of the Himalayan marmot in the study regions. While small mammals generally account for lower levels of biomass than larger mammals, resulting in a relatively small contribution to dung fungal spore production [56], their contribution may become more substantial in the absence of large herbivores. The coprophilous fungal spores found in marmot faeces resemble those typically associated with larger herbivores. Therefore, when interpreting palaeoecological fungal spore records in mountainous regions where large herbivore densities are low, the potential contribution of smaller mammals such as marmots should not be overlooked.

Among the non-coprophilous fungal spores, *Thecaphora* is present with average values of 16.6% and 11.3% in both areas respectively. *Thecaphora* sp. is known to infect *Silene* (Caryophyllaceae) [57] plants which can be incorporated into the faeces through the ingestion of the infected plants. The clumping of *Thecaphora* is observed in samples from both areas and is strongly indicative of the local origin of these spores. The other non-coprophilous fungal spores, *Helminthosporium*, *Alternaria*, and *Meliola*, are also regularly present in the assemblages which can also be incorporated into the faeces through the ingestion of infected leaves.

### Cross-regional similarities in marmot diet in relation to the dietary plants

The analysis yielded Eigen values of 0.2463 (Axis 1) and 0.1309 (Axis 2), explaining a combined variance of 58.31% based on pollen taxa identified in faeces samples from the Pensila Pass (Ladakh) and Rimkhim region (Uttarakhand) (Fig 6). Our PCA results suggest that the differences between the two study areas are minimal, typically amounting to only few percentage points. The relative abundance of dietary plant pollen between the two locations sampled indicates Asteroideae, Poaceae, Chenopodiaceae, and *Ephedra* are relatively more abundant in the Pensila Pass area, while *Artemisia*, Caryophyllaceae, and Cyperaceae are relatively higher in abundance in the Rimkhim area (Fig 6). Despite these minor differences, the pollen assemblages from the two distant locations indicate a consistent dietary preference among Himalayan marmots, with only slight differences in the proportion of consumed plant taxa. The pollen data from the marmot faeces samples of both regions could also be utilized as an auxiliary tool for developing a modern pollen analogue from the higher Himalayan region, complementary to pollen records from the dung of wild Yak that lives in the Indian Himalaya [25].

### Pollen ratio and its implication to the palaeoecological analysis

Some researchers have utilized the ratio values of pollen especially *Artemisia*/Chenopodiaceae (A/Ch), *Artemisia*/Cyperaceae (A/Cy), and *Artemisia*/*Ephedra* (A/Ep) to interpret the vegetation types as indicator taxa and climate at higher altitudes based on analysis of pollen assemblages in the dust and soil samples [55–65]. The modern pollen study from the soil samples of the Tso Kar lake (Ladakh), which is approximately 300 kms from the Pensilla Pass, had a ratio of A/Ch is 2.6 [64], compared to >2 dust flux samples for the steppe and sub desert vegetation in the Karakorum region [63] and is approximately 110 kms from the Pensilla Pass. The A/Ch ratio of the surface soil sample is about 1 in the Pangong Co region, which is characterized by high-Alpine Tibetan desert vegetation [60]. In Kashi-Yecheng, the A/Cy ratio is < 1, with annual precipitation <100 mm; the A/Cy ratio rose to 1–2 (1.42 average) with precipitation between 50–100 mm [63]; (<1-Saline desert; 1–2-Montane desert; > 2 Montane steppe). Similarly, the A/Cy ratio can be used to differentiate between meadow (<1) and steppe (>1) in the southwestern Tibetan Plateau [63,65,66]. Changes in the A/Ep ratio can serve as an indicator of glacial and interglacial phases [58]. Additionally, the pollen ratio between *Artemisia* and Chenopodiaceae serves as an effective indicator of moisture variability, with *Artemisia* being more prevalent under relatively more humid conditions [59–65].

Our data show ratio values of A/Ch, A/Cy, and A/Ep, from the Pensilla Pass and Rimkhim areas recorded in the pollen assemblages as an index value to understand the extant vegetation and climate in relation to the marmot faeces sample (S3 Table 3). The ratio values of A/Ch from Pensilla Pass are 2.6% and Rimkhim region 2.3%, which is similar to that of dust samples in the Karakorum and soil sample of Tso kar region (Ladakh). Similarly, the ratio values of A/Cy from the Pensilla Pass and Rimkhim regions is 3.86% and 3.9% respectively, which again is similar with the soil samples of the southwestern TP. Subsequently, the A/Ep ratio values from the Pensilla Pass and Rimkhim region is 40.1% and 44.2% respectively and differs from other sites which dust sample in the Kunlam (13.89%), Tian Shi hai (12.4%), and Taklimakan (6.41%). Moreover, the recorded values of pollen ratio confirm the marmot faeces samples also have the potential for the interpretation of the vegetation and climate in higher Himalaya. The recorded values from the faeces sample are also a useful and complementary to the surface soil samples as evidence of the comparative analysis data of the Tibet plateau and neighbouring areas (S3 Table 3). Based on the ratio values of obtained from this study, we have characterised that there is minimal significant differences in pollen ratio values between the samples from the Pensilla Pass and Rimkhim area and support the interpretation that the extant vegetation and climate in the region can serve as evidence of the representation of the major pollen taxa. However, the ratio value A/Ep, in the Rimkhim areas points to slightly lower aridity compared to the Pensila Pass region.

### Nutritional elements in marmot faeces and its implication for modern faeces and coprolite studies

The use of FESEM-EDS analysis allowed us to determine the nutritional elements present in the Himalayan marmot faeces, so can provide insights into the nutritional value of their dietary plants. This modern generated data can also serve as a baseline for the fossil data analysis which this the elemental study of the modern faeces record will be helpful for interpretation of palaeodietary analysis.

A total of 15 nutrient elements were examined in this study (S1 Fig. 1; S1 Table 1 and S2 Table 2). The results reveal notably high concentrations of calcium and phosphorus in the samples. Given that *Artemisia*, which is rich in both calcium and phosphorus [67,68] has been identified as a primary dietary component of the Himalayan marmot, it is likely the principal source of these elements in the faeces. Furthermore, a flower-rich diet is known to contribute to fat deposits, fatty acids, crude proteins, and elevated phosphorus levels [69].

The other elements, N, Si, Al, K, Fe, and Cu are also regularly present in the faeces sample. A similar study has been documented on the wild yak from the higher Himalayas [25] and reveals that there are some differences in the frequency of the elemental content. The difference of the frequency might be due to the variation of dietary plants. So, by combining this elemental record from the modern Himalayan marmot faeces samples is helpful and complementary with the biological proxies during the identification and characterization about the other modern faeces of herbivores and coprolites analysis. However, our data provide some important information about the nutritional elements in relation to the dietary plants for the Himalayan marmot survival in the higher Himalayan region. The analysis of pollen, fungal spores, and elemental data can serve as a baseline for the coprolite and palaeoenvironmental interpretation in relation to the current vegetation and climate in the Himalayan marmot habitat regions.

### Marmot faeces analyses as bioindicator for the past and future environmental studies

The Himalayan marmot is a high-altitude and primarily inhabiting with the lower limit of its range between 4200–5200 m asl which in between the timberline and snowline [70–72]. The Himalayan marmot is likely restricted to higher elevations because its physiological adaptations to cold temperatures and hypoxic conditions limit its survival at lower altitudes [73,74]. Moreover, temperatures in its habitat rarely exceed the critical threshold of 22 °C −25 °C, above which marmots reduce their surface activity [75,76]. Marmot faeces preserve pollen, phytoliths, and plant remains in relation to their dietary plants, making them a reliable bioindicator of local vegetation and environmental conditions.During the Pleistocene period, the Himalayan glaciers extended to lower altitude ranging between 1678–1830 m asl, suggesting that marmots likely occupied lower altitudes under colder climates [77,78]. Recent climate change studies reveal that the Himalaya is warming exponentially with an annual temperature increase of 0.06° C, compared to the global average of 0.02° C [79]. Moreover, in the western Himalayan region including parts of India, Pakistan and Afghanistan, the temperature rose by approximately 0.46° C per decade between 1971 and 2007 [80], where Ladakh has experienced a particularly sharp increase in summer temperatures over the past thirty years [81]. These climate changes have already begun to alter mountain ecosystems, with the treeline shifting approximately 400 m upward over the past 60 years, resulting in the loss of over one-quarter of forest area [82]. Subsequent warming led to the upward shift of glaciers, treelines, and alpine habitats, forcing marmot populations to migrate to higher altitude. Thus, faeces analysis offer a robust tool to reconstruct palaeoenvironments, track ongoing vegetation shifts, and infer the implications of climate change on the Himalayan ecosystem.

## Conclusions

This study presents pollen and fungal spore data from the Pensilla Pass and Rimkhim region of the higher Himalaya accompanied by nutrient composition from recent Himalayan marmot faeces, suggesting that faeces serve as records of modern environmental and climatic conditions that could be extrapolated to the fossil record. Our data revealed a mixed plant-based diet dominated by *Artemisia*, Asteroideae, Caryophyllaceae, and Chenopodiaceae in relation to the modern Alpine steppe vegetation under cold and arid climate in the higher Himalayan region. This study also confirmed that the Himalayan marmot has a direct relationship between the vegetation type and climate along with the altitudinal range in the higher Himalayan regions. The long term biotic and abiotic studies on marmot faeces in the marmot habitat areas of higher Himalayas will be helpful to trace the important factors for the shifting of marmot habitat in relation to snowline in the higher Himalaya. For the first time, pollen ratios between *Artemisia*/Chenopodiaceae, *Artemisia*/Cyperaceae, *Artemisia*/*Ephedra* from marmot faeces samples have been calculated and identified as a significant values useful for the interpretation of past vegetation and climate changes in the higher Himalaya. Additionally, after analysis of the elemental composition of the marmot faeces samples offers insight into the nutrient elements in relation to the primary dietary plants which will be enhanced our understanding of palaeodietary analysis and potential conservation concerns. After analysing the detailed of the pollen and NPPs from habitat of the Himalayan marmot in the altitudinal ranges, we hypothesized that the Himalayan marmot emerges as a valuable bioindicator for tracking vegetation and climate changes in the higher Himalayas in future studies.

## Supporting information

S1 Table 1: List of the elements value generated by FESEM-EDS analysis in *Marmota himalayana* faeces sample collected from Pensila Pass area. (PDF)

S2 Table 2. List of the elements value generated by FESEM-EDS analysis in *Marmota himalayana* faeces sample collected from Rimkhim area. (PDF)

S3 Table 3: Characterization of *Artemisia*/Chenopodiaceae, *Artemisia*/Cyperaceae, and *Artemisia*/*Ephedra* pollen ratio value recorded from the Himalayan marmot faeces in relation to the vegetation types and different substrate analysis from Tibet plateau and neighbouring areas. (PDF)

S1 Fig Micrographs of FESEM-EDS elemental analysis in *Marmota himalayana* faeces samples collected from Pensila Pass (a) and Rimkhim (b). (PDF)
