# Supplementary material for: Modern Himalayan marmot faeces analyses: Implications for palaeodietary, palaeoecological, and palaeoclimate analysis
Source: PLoS One. 2026 Oct 28;21(10):e0358642. doi: 10.1371/journal.pone.0358642 (PMC13716175; doi:10.1371/journal.pone.0358642)
Supplement: Supplementary file 1 [file pone.0358642.s001.pdf]

S1 Table 1. List of the elements value generated by FESEM-EDS analysis in *Marmota himalayana* faeces sample collected from Pensila Pass area.

Element	Weight %
C	20.11
N	7.78
O	45.74
Na	0
Mg	0.97
Al	1.55
Si	4.23
P	3.82
S	0.56
K	3.74
Ca	8.48
Mn	0.32
Fe	1.45
Co	0.31
Ni	0.24
CuK	0.71
