# Supplementary material for: Modern Himalayan marmot faeces analyses: Implications for palaeodietary, palaeoecological, and palaeoclimate analysis
Source: PLoS One. 2026 Oct 28;21(10):e0358642. doi: 10.1371/journal.pone.0358642 (PMC13716175; doi:10.1371/journal.pone.0358642)
Supplement: Supplementary file 2 [file pone.0358642.s002.pdf]

S2 Table 2. List of the elements value generated by FESEM-EDS analysis in *Marmota himalayana* faeces sample collected from Rimkhim area.

Element	Weight %
C	24.68
N	4.75
O	48.71
Na	0
Mg	0.51
Al	0.7
Si	2.71
P	4.11
S	0.78
K	1.94
Ca	6.84
Mn	0.5
Fe	1.66
Co	0.45
Ni	0.45
Cu	1.21
