# Supplementary material for: Modern Himalayan marmot faeces analyses: Implications for palaeodietary, palaeoecological, and palaeoclimate analysis
Source: PLoS One. 2026 Oct 28;21(10):e0358642. doi: 10.1371/journal.pone.0358642 (PMC13716175; doi:10.1371/journal.pone.0358642)
Supplement: Supplementary file 3 [file pone.0358642.s003.pdf]

S3 Table 3: Characterization of *Artemisia*/Chenopodiaceae, *Artemisia*/Cyperaceae, and *Artemisia*/*Ephedra* pollen ratio value recorded from the Himalayan marmot faeces in relation to the vegetation types and different substrate analysis from Tibet plateau and neighbouring areas.

Study region	Vegetation type/rainfall	Substrate	A/Ch	A/Cy	A/Ep	References
Pangong Co region	High alpine desert	Surface soil	1	-	-	Van Campo et al. 1996
Karakorum	Montane steppe and sub-desert/100-800 mm	Dust flux pollen	2.22	-	34.47	Cour et al. 1999
Kunlam	Montane desert and alpine desert steppe/50-400 mm	Dust flux pollen	1.42	-	13.89	Cour et al. 1999
Tian Shui hai	Alpine desert steppe/50-100 mm	Dust flux pollen	1.38	-	12.4.	Cour et al. 1999
Taklimakan	Sand-gravelly desert and saline desert steppe/20-100 mm	Dust flux pollen	0.36	-	6.41	Cour et al. 1999
Central and northeastern TP	Alpine steppe	Soil	-	1	-	Herzschuh 2007
Tso Kar (Ladakh)	Alpine Steppe and desert steppe/100-300 mm	Soil	2.6	-	-	Demske et al. 2009
Southwestern TP	Sub-alpine steppe/50-500 mm	Soil	-	3.9	-	Ma et al. 2017
Pensila Pass (Ladakh)	Alpine Steppe	Himalayan marmot faeces	2.6	3.86	40.1	Basumatary et al.
Rimkhim (Uttrakhand)	Alpine Steppe	Himalayan marmot faeces	2.3	3.9	44.2	Basumatary et al.
