# Supplementary material for: Modern Himalayan marmot faeces analyses: Implications for palaeodietary, palaeoecological, and palaeoclimate analysis
Source: PLoS One. 2026 Oct 28;21(10):e0358642. doi: 10.1371/journal.pone.0358642 (PMC13716175; doi:10.1371/journal.pone.0358642)
Supplement: Supplementary file 4 [file pone.0358642.s004.pdf]

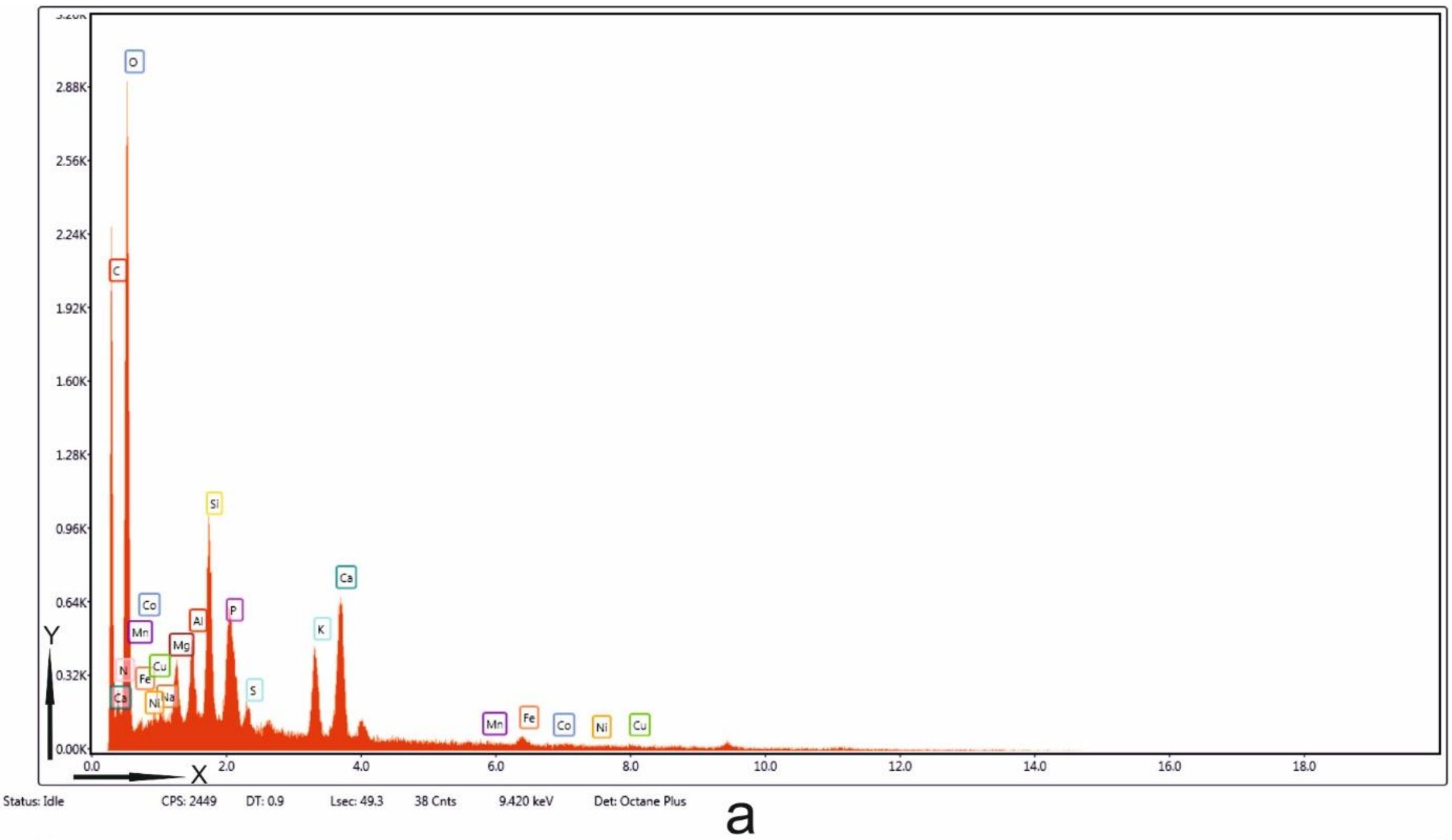

a

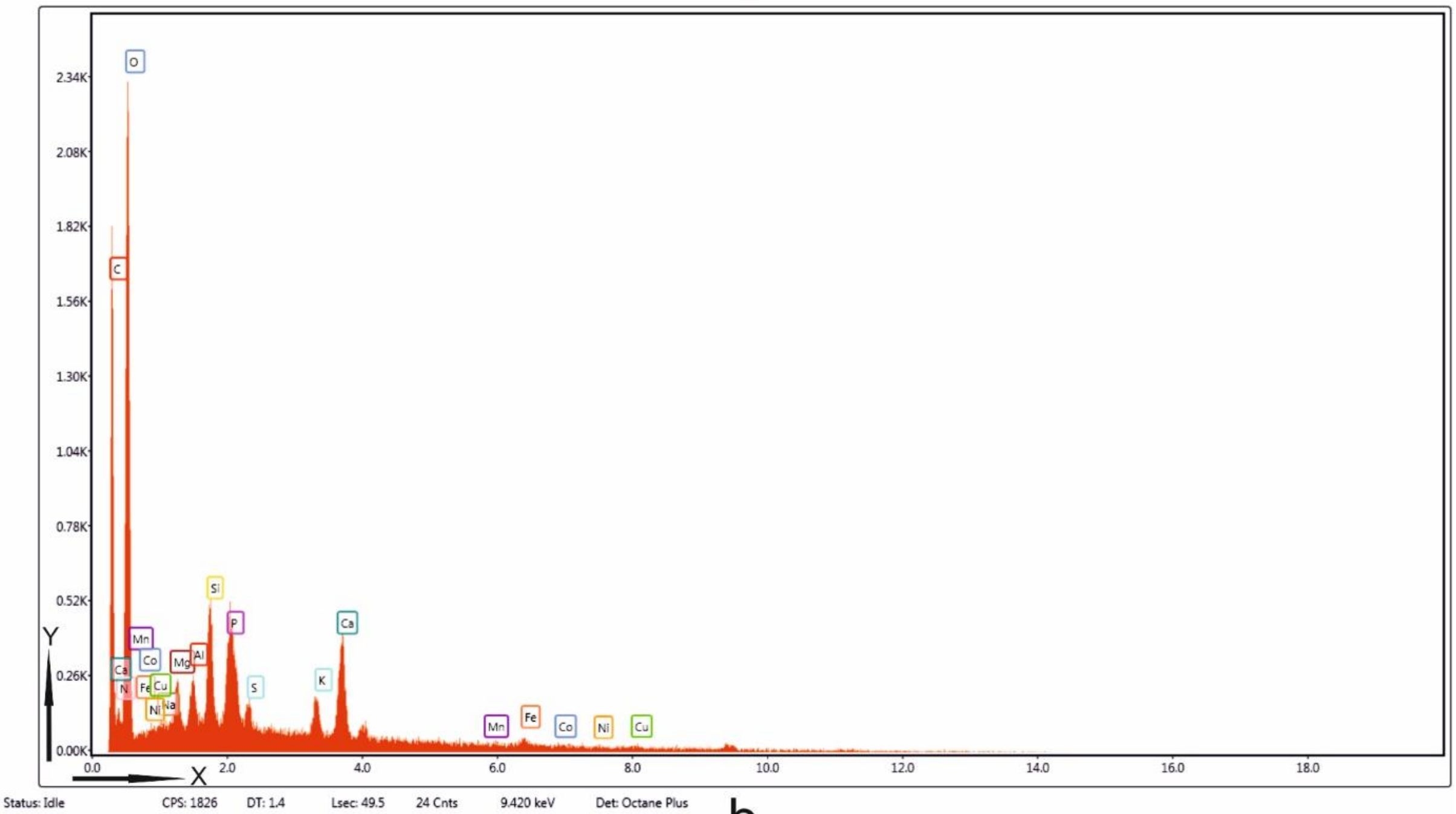

b

LEGENDS-

X axes: Energy (kilo electron volt); Y axes: Intensities
